# Personalized neoantigen-pulsed dendritic cell vaccines show superior immunogenicity to neoantigen-adjuvant vaccines in mouse tumor models

**DOI:** 10.1007/s00262-019-02448-z

**Published:** 2019-12-05

**Authors:** Rui Zhang, Fengjiao Yuan, Yang Shu, Yaomei Tian, Bailing Zhou, Linglu Yi, Xueyan Zhang, Zhenyu Ding, Heng Xu, Li Yang

**Affiliations:** 1 State Key Laboratory of Biotherapy and Cancer Center/Collaborative Innovation Center for Biotherapy, West China Hospital, No. 17, Section 3, South Renmin Road, Wuhou District, Chengdu, 610041 Sichuan China; 2 State Key Laboratory of Biotherapy, and Precision Medicine Key Laboratory of Sichuan Province, Precision Medicine Center, West China Hospital, Sichuan University and Collaborative Innovation Center, Chengdu, China; 3 State Key Laboratory of Biotherapy, West China Medical School, Cancer Center, West China Hospital, Sichuan University, Chengdu, China

**Keywords:** Personalized neoantigens, Neoantigen-pulsed DC vaccines, Neoantigen-adjuvant vaccines, Immune response, Anti-tumor

## Abstract

Development of personalized cancer vaccines based on neoantigens has become a new direction in cancer immunotherapy. Two forms of cancer vaccines have been widely studied: tumor-associated antigen (including proteins, peptides, or tumor lysates)-pulsed dendritic cell (DC) vaccines and protein- or peptide-adjuvant vaccines. However, different immune modalities may produce different therapeutic effects and immune responses when the same antigen is used. Therefore, it is necessary to choose a more effective neoantigen vaccination method. In this study, we compared the differences in immune and anti-tumor effects between neoantigen-pulsed DC vaccines and neoantigen-adjuvant vaccines using murine lung carcinoma (LL2) candidate neoantigens. The enzyme-linked immunospot (ELISPOT) assay showed that 4/6 of the neoantigen-adjuvant vaccines and 6/6 of the neoantigen-pulsed DC vaccines induced strong T-cell immune responses. Also, 2/6 of the neoantigen-adjuvant vaccines and 5/6 of the neoantigen-pulsed DC vaccines exhibited potent anti-tumor effects. The results indicated that the neoantigen-pulsed DC vaccines were superior to the neoantigen-adjuvant vaccines in both activating immune responses and inhibiting tumor growth. Our fundings provide an experimental basis for the selection of immune modalities for the use of neoantigens in individualized tumor immunotherapies.

## Introduction

Vaccination is one of the most effective ways to stimulate the body’s immune response. Currently, one of the most popular approaches of cancer immunotherapy is to screen neoantigens, which arise from altered proteins due to genomic mutations, in patients, and then present the antigens to immune cells through vaccination to activate T-cell responses and kill tumor cells, thus achieving precision treatment [1–3]. Peptide vaccines have been extensively studied, because they are simple, safe, and economical [4]. However, due to their unique peptide epitopes, low molecular weights, easy degradation, and short half-lives, peptide vaccines have two basic limitations: low immunogenicity and major histocompatibility complex (MHC) restriction [5, 6]. Therefore, adding immunological adjuvants to peptide vaccines is essential to induce an effective immune response [7]. The oil-in-water emulsion adjuvants and aluminium adjuvants are more widely used and prove effective for cancer vaccines, although other types of adjuvants have also achieved good anti-tumor effects [8, 9]. In addition, to improve the anti-tumor effects of neoantigens, DC-based tumor vaccines have been increasingly studied [10–14]. Their therapeutic effects are efficient, and the side effects are minimal. DC is the most potent antigen-presenting cells (APCs) with the ability to stimulate immune cells and memory effector cells [15]. Since the first use of melanoma-associated antigen (MAGE1)-loaded DC in 1995 to treat melanoma in vitro, more than 400 clinical trials based on DC vaccines have been conducted or completed for the treatment of various malignant tumors [15–17].

Recently, neoantigen-adjuvant vaccines and neoantigen-pulsed DC vaccines have been extensively studied in clinical trials. Both of them exhibit good anti-tumor effects in clinical trials [10–12]. However, different vaccine strategies may produce different therapeutic effects when the same antigen is used. Therefore, choosing effective immunization approaches is critical to acquire a strong and long-lasting immune response. A previous clinical trial has shown that treatment of metastatic melanoma with antigen-adjuvant vaccines only exhibits an objective tumor decline rate of 2.7%, whereas treatment of metastatic melanoma with a DC vaccine achieves an objective regression rate of 9.5% [18]. The results suggest that antigen-pulsed DC vaccines may have advantages over antigen-adjuvant vaccines. To our knowledge, no research has been conducted to evaluate the anti-tumor effects of antigen-pulsed DC vaccines and antigen-adjuvant vaccines using the same antigen. Additionally, further work is needed to clarify why antigen-pulsed DC vaccines are superior to antigen-adjuvant vaccines.

This study was set to evaluate the immune and anti-tumor effects of the neoantigen-pulsed DC vaccines and the neoantigen-adjuvant vaccines using murine lung carcinoma (LL2) candidate neoantigens. To understand the mechanism by which the antigen-pulsed DC vaccines are superior to the antigen-adjuvant vaccines, we further determined their effects on the immunogenicity, anti-tumor factors, and cytokine levels in the serum, the proportions of activated splenic CD8^+^ T cells and CD44^+^ CD62L^+^ memory T cells, and the proportions of infiltrating T cells and inhibitory T cells in the tumor microenvironment in mouse LL2 xenograft models.

## Methods

### Cell culture conditions

Mouse Lewis lung carcinoma LL2 cells (American Type Culture Collection, Manassas, VA, USA) were cultured in Dulbecco’s modified Eagle’s medium (DMEM) supplemented with 10% fetal bovine serum (FBS) and 100 units/ml penicillin (P)/100 μg/ml streptomycin (S). All cells were cultured in a humid incubator (37 °C and 5% CO_2_) and trypsinized with 0.05% trypsin-ethylene diamine tetraacetic acid (EDTA) for subculture or experiments. DMEM, FBS, trypsin–EDTA, and PS were all purchased from Thermo Fisher Scientific (Waltham, MA, USA).

### Whole-exon sequencing of mouse tumor tissues and analysis of tumor-specific neoantigens

Female C57BL/6 J mice (8–10 weeks old) were implanted subcutaneously on the right flank with 1 × 10^6^ LL2 tumor cells. In 2 weeks, the tumors were dissected and the whole blood was collected in triplicate, followed by extraction of whole-genomic DNA (*n* = 3) using QIAamp DNA Mini Kit (Qiagen, Shanghai, China) and RNA (*n* = 3) using QIAamp DNA Blood Mini Kit (Qiagen, Shanghai, China). Then, whole-exon and transcriptome sequencings were carried out on an Illumina HiSeq 2000 (Illumina, Shanghai, China).

Tumor-specific neoantigens were selected through the following steps: (1) removal of low-quality reads through a filtering step [19]; (2) detection of single-nucleotide variants with the Mutect and VarScan methods [20, 21]; (3) use of ANNOVAR software to annotate missense mutations [22]; (4) adoption of a sliding window protocol to extract the peptides containing mutant (MUT) amino acid residues and their corresponding wild-type (WT) peptides from the protein sequence (note: a window with an interval of one amino acid was applied nine times, so that nine different peptide sequences with mutated sites from the first amino acid to the ninth amino acid were obtained, and the same peptide sequences corresponding to the gene fragment of the normal mouse were simultaneously extracted); (5) use of the PSSMHCpan algorithm to evaluate the MHC I affinity of the tumor neoantigens and the corresponding WT sequences [23]; (6) analysis of neoantigen expression levels in the transcriptome; and (7) through exon sequencing and bioinformatic analysis of the mouse LL2 lung cancer cell line. The screened and synthesized six MHC class I neoantigens are shown in Table 1. All peptides (≥ 95% purity) were synthesized by GL Biochem (Shanghai, China).

**Table 1:** List of candidate neoantigens of MHC I in LL2 lung cancer cell line

Gene-MUT	MUT/WT sequence	IC_50_ (MUT/WT)
*Mtmr10*_R633I	FS(I/R)PANLHGI	59/2692
*Elfn2*_P762L	LSPRHYYSGYSSS(L/P)	45/2631
*Kat8*_P448L	VCLKWAP(L/P)	53/15887
*Mastl*_D366Y	LSPIH(Y/D)SSA	302/12194
*Zscan21*_H409L	LTLHYRT(L/H)	56/22121
*Mrpl1*_C32F	SLYP(F/C)SVNSL	146/2248

*MUT* mutant, *WT* wild type

### Preparation of neoantigen-pulsed DC vaccines and neoantigen-adjuvant vaccines

To perform the antigen-pulsed DC vaccination, DCs derived from bone marrow progenitor cells were obtained as previously reported [13]. Briefly, bone marrow primary cells were cultured for 8 days in RPMI 1640 medium (Thermo Fisher Scientific) with 10% FBS, PS, and granulocyte–macrophage colony-stimulating factor (GM-CSF) (20 ng/ml) (Prime Gene Biotechnology, Shanghai, China). On day 7, the selected neoantigens (10 μg/ml) were added to the immature DCs for 24 h, which were then stimulated with lipopolysaccharide (LPS; 1 μg/ml; Beyotime Biotechnology, Shanghai, China), CPG (10 μg/ml) (Invitrogen, Carlsbad, CA, USA), and interferon gamma (IFN-γ) (50 ng/ml; Prime Gene Biotechnology) for 24 h to obtain mature DCs. The mature neoantigen-loaded DCs were then harvested, counted, and resuspended in serum-free RPMI 1640 medium. To perform the neoantigen-adjuvant vaccination, 100 μg of the selected neoantigens were thoroughly mixed with complete Freund’s adjuvant (CFA) or incomplete Freund’s adjuvant (IFA).

### Detection of neoantigen immunogenicity

For ELISPOT assay, all mice were sacrificed 1 week after the completion of the last immunization and spleen lymphocytes were harvested for experiments. The ELISPOT assay was performed as previously reported [13]. Briefly, 5 × 10^5^ mouse splenocyte lymphocytes were seeded in a 96-well microtiter plate pre-coated with anti-IFN-γ antibody. Next, 10 μg/ml of wild-type peptides or mutant peptides were added and the plate was incubated at 37 °C. After 72 h, the culture broth was discarded from the wells and pre-cooled ddH_2_O was added at 4 °C for 10 min to lyse the cells in the plate. The plate was then washed five times with wash buffer. Next, the diluted biotin-labelled secondary antibody was added to each well followed by incubation for 1 h at 37 °C. For the enzyme-linked avidin incubation, the diluted avidin enzyme working solution was added to each test well and the plates were incubated at 37 °C for another 1 h. The prepared aminoethyl carbazole solution was then added and the colour reaction was allowed to occur at 37 °C in the dark for approximately 10 min. Finally, the plates were photographed and read using Bio-Reader 4000 (Byosys, Karben, Germany).

For flow cytometry analysis, all mice were sacrificed 1 week after the completion of immunization, and spleen lymphocytes were harvested as described above. A total of 3 × 10^6^ spleen lymphocytes were suspended in 600 μL of RPMI 1640 medium-containing 10% FBS and PS and added to 6-well plates. Each group was stimulated with 10 μg/ml WT peptides or neoantigens at 37 °C for 12 h. The cells were then collected and the proportion of cytotoxic T lymphocytes was detected by staining with anti-mouse-CD3, anti-mouse-CD8, and anti-mouse-IFN-γ fluorescent antibodies (BD Biosciences, San Jose, CA,USA).

### Assessment of the anti-tumor effects and the changes of tumor microenvironment in mice immunized with neoantigen-pulsed DC vaccines and neoantigen-adjuvant vaccines

A total of 2 × 10^6^ DCs were injected intravenously into C57BL/6 J mice twice every 2 weeks. One week after the last immunization, 1 × 10^6^ LL2 cells were implanted subcutaneously into the right flank of each mouse. All mice were sacrificed on the 17th day post-implantation. To perform the neoantigen-adjuvant vaccination, C57BL/6J mice were subcutaneously injected with each antigen-adjuvant vaccine thrice as follows: on day 0, with 100 μg of peptide with CFA; on day 14 and day 28, both with 100 μg of peptide with IFA. Then, LL2 cells were implanted to mice, which were sacrificed 17 days post-implantation, as described above. The tumor sizes were recorded every other day. Once the mice were sacrificed, tumor tissues were harvested and stained with anti-mouse-CD45, anti-mouse- CD3, anti-mouse-CD8, and anti-mouse-IFN-γ fluorescent antibodies to determine the percentage of positive cells (cytotoxic T lymphocyte, CTL).

### Evaluation of the immune responses of mice immunized with neoantigen-pulsed DC vaccines and neoantigen-adjuvant vaccines

Among the six initially identified neoantigens, Elfn2_P762L and Mastl_D366Y were selected to evaluate the differences in the additional immune responses induced by two immune modalities. Elfn2_P762L was selected, because it showed significant differences compared with the control groups in the ELISPOT results in the two vaccine forms. However, Elfn2_P762L-adjuvant vaccine was ineffective, while Elfn2_P762L-pulsed DC vaccine was effective, in terms of anti-tumor activity. Mastl_D366Y was selected, because it was effective both in Mastl_D366Y-pulsed DC vaccine and Mastl_D366Y-adjuvant vaccine, with regard to the anti-tumor activity. Immunizations were carried out as described above. Briefly, tumor cells were implanted to mice 1 week after the last immunization, and the mice were sacrificed 17 days post-implantation. After the death of the animals, tumor tissues, spleens, and draining lymph nodes were immediately collected for further study. To determine the memory T-cell responses, spleen lymphocytes were stained with anti-mouse-CD3, anti-mouse-CD8, anti-mouse-CD44, and anti-mouse-CD62L fluorescent antibodies, followed by flow cytometry analysis. To examine the number of T cells infiltrating into the tumor microenvironment, tumor tissues were digested to single cell suspensions with collagenase IV. Then, the cell suspensions were stained with anti-mouse-CD45, anti-mouse-CD3, anti-mouse-CD8, and anti-mouse-IFN-γ fluorescent antibodies to determine the percentage of CTL. The tumor tissues were then stained with anti-mouse-CD45, anti-mouse-CD3, anti-mouse-CD4, and anti-mouse-Foxp3 fluorescent antibodies to determine the percentage of regulatory T cells. Similarly, to detect DC activation in the draining lymph nodes, cells were collected from lymph-node homogenates and stained with anti-mouse-CD11c, anti-mouse-CD86, and anti-mouse-CD80 fluorescent antibodies. Flow cytometry was then used to analyze the percentage of CD11c^+^CD80^+^CD86^+^ DCs in the draining lymph nodes. All these antibodies were purchased from BD Biosciences.

One week after the last immunization, blood was taken from the retro-orbital sinus of mice. Serum was separated by centrifugation at 13,000 rpm for 20 min. IL-6, IL-10, and IL-12p70 ELISA kits (Novus, Centennial, CO, USA) and TNF-α ELISA kit (abcam, Cambridge, UK) were used to detect the serum levels of corresponding cytokines, according to the vendors’ instructions.

### Histological analysis

Tumor tissues and major organs obtained from in vivo studies were immediately fixed with 4% paraformaldehyde and then embedded in paraffin. The embedded tissue sections were deparaffinized and rehydrated before staining. For immunofluorescence analysis, antigens in tumor sections were recovered under high pressure and then incubated with anti-mouse-CD8 and anti-mouse-Foxp3 fluorescent antibodies (BD Biosciences) for 1 h at 4 °C. Sections were evaluated and images were captured under aDM 2500 fluorescence microscope (Leica, Wetzlar, Germany) equipped with a digital camera.

### Statistical analysis

Statistical analyses were performed using a two-tailed *t* test or one-way analysis of variance using Prism 6.0 software (San Diego, CA, USA). Statistical significance was defined by a value of *P *< 0.05.

## Results

### The neoantigen-pulsed DC vaccines elicit a stronger antigen-specific lymphocyte response than the neoantigen-adjuvant vaccines

**Fig. 1:**
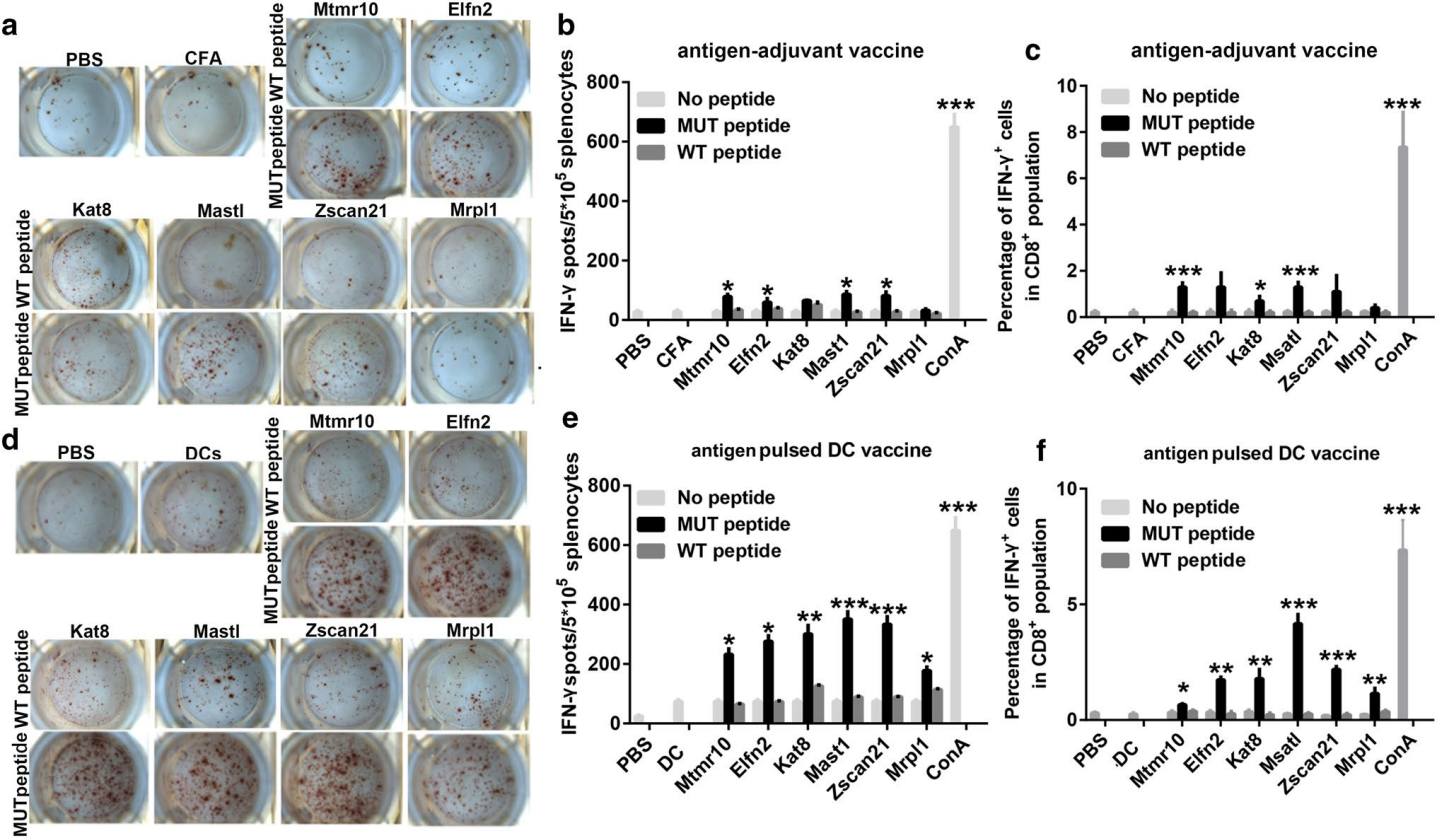
Detection of antigen-specific splenic lymphocyte responses by ELISPOT and flow cytometry analysis. Mice were immunized with the neoantigens combined with Freund’s adjuvant or the neoantigen-pulsed DC. 1 week after the last immunization, spleen lymphocytes were isolated and stimulated with neoantigens or their wild-type peptides to observe the antigen-specific T-cell responses (*n* = 3); **a**, **b** Images and graphical representation of the ELISPOT results from the antigens combined with Freund’s adjuvant groups. **c** Percentages of the CD8^+^IFN-γ^+^ spleen lymphocytes in the neoantigen-adjuvant vaccine groups. **d**, **e** Intuitive diagrams and statistical charts of the ELISPOT results from the neoantigen-pulsed DC vaccine groups; **f** Percentages of the CD8^+^IFN-γ^+^ spleen lymphocytes in neoantigen-pulsed DC vaccine groups. *P < 0.05, **P < 0.01, and ***P < 0.001

To evaluate the immunogenicity of the neoantigens shown in Table 1, female C57BL/6J mice were immunized with the selected neoantigens using two forms of vaccine, neoantigen-pulsed DC vaccines, and neoantigen-adjuvant vaccines. One week after the last vaccination, ELISPOT and flow cytometry were performed to detect neoantigen-specific spleen lymphocyte responses. The ELISPOT results showed that 4/6 of the neoantigen-adjuvant vaccines (MUT-Mtmr10, MUT-Elfn2, MUT-Msatl, and MUT-Zscan21) induced a significantly increased secretion of IFN-γ compared to the PBS and adjuvant-alone groups (Fig. 1a, b). Similarly, the flow cytometry results showed that the MUT-Mtmr10, MUT-Kat8, and MUT-Msatl neoantigen-adjuvant vaccines induced a significantly increased proportion of neoantigen-specific CD8^+^ IFN-γ^+^ T cells in the spleen (*P *< 0.001) (Fig. 1c). Of interest, 6/6 of the selected neoantigen-pulsed DC vaccine induced a significantly increased secretion of IFN-γ by spleen lymphocytes, as detected by ELISPOT assay (Fig. 1d, e). In line with this, the flow cytometry analyses also showed that 6/6 of the neoantigen-pulsed DC vaccines induced a significantly increased release of IFN-γ from neoantigen-specific CD8^+^ T cells (Fig. 1f). Moreover, all of the vaccine groups did not respond to wild-type peptide stimulation, suggesting that the activated T-cell response was neoantigen specific.

### The neoantigen-pulsed DC vaccines inhibit tumor growth and increase the number of tumor infiltrating lymphocytes more efficiently than the neoantigen-adjuvant vaccines

**Fig. 2:**
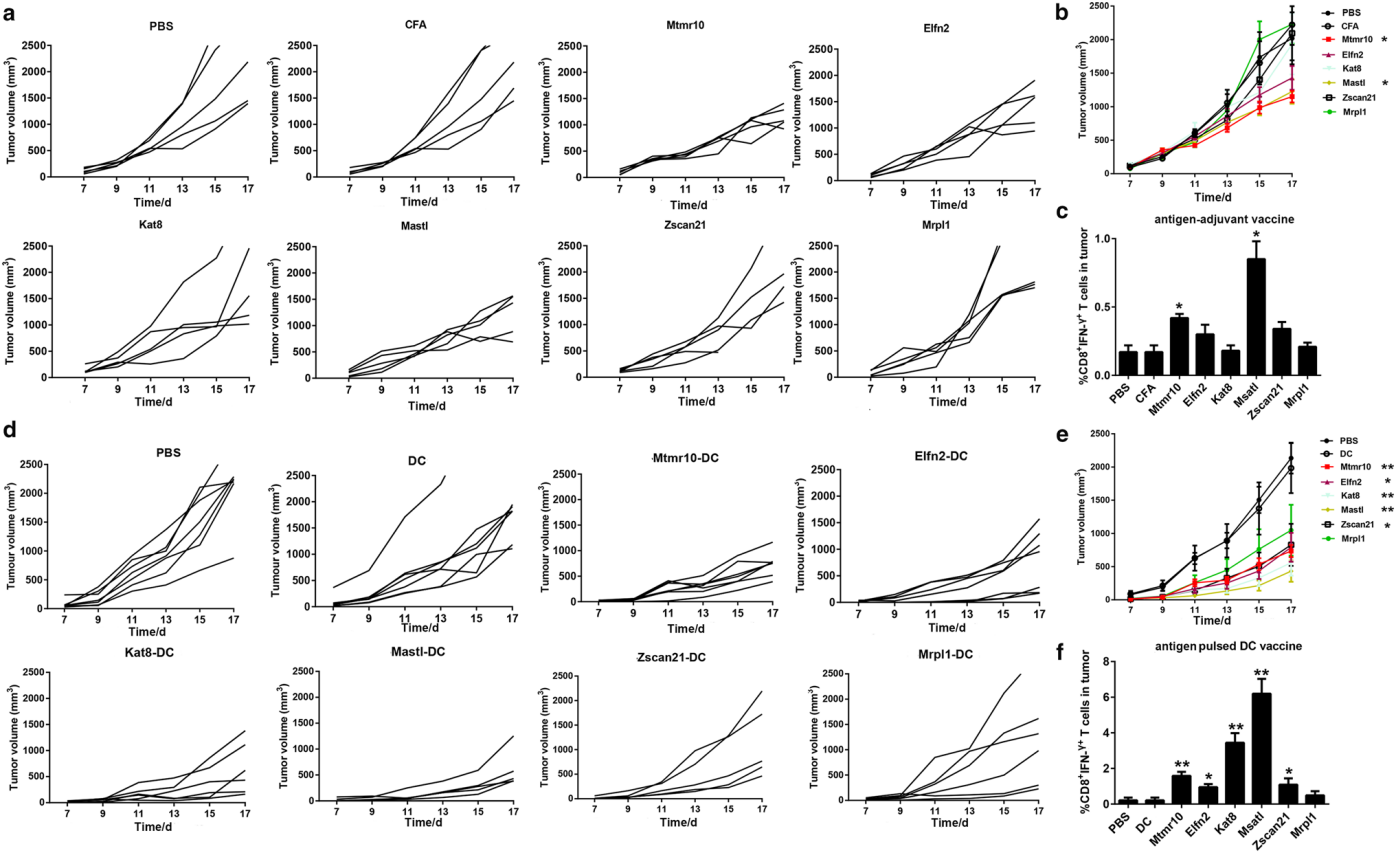
Anti-tumor effects of the neoantigen-adjuvant vaccines and neoantigen-pulsed DC vaccines. **a** Tumor growth curves for each mouse in the neoantigen-adjuvant vaccine groups. **b** Mean tumor volumes in the neoantigen-adjuvant vaccine groups. **c** Histogram of the percentage of CD8^+^ IFN-γ^+^ CTL in tumors from the neoantigen-adjuvant vaccine groups. **d** Tumor growth curves of each mouse in the neoantigen-pulsed DC vaccine groups. **e** Means of the tumor volumes in the neoantigen-pulsed DC vaccine groups. **f** Histogram of the percentage of CD8^+^ IFN-γ^+^ CTL in tumors of the neoantigen-pulsed DC vaccine groups. *P < 0.05, **P < 0.01

To check whether the neoantigen-pulsed DC vaccines exhibit a better anti-tumor activity, the LL2 subcutaneous xenograft model was employed. We found that the tumor growth was significantly inhibited in the mice treated with the MUT-Mtmr10 and MUT-Msatl neoantigen-adjuvant vaccines, but not with other four neoantigen-adjuvant vaccines, compared to that of the PBS and adjuvant-alone treatment groups (*P *< 0.05) (Fig. 2a, b). Consistently, the infiltration of CD8^+^IFN-γ^+^ T cells was significantly increased in 2/6 tumors from the neoantigen-adjuvant vaccine groups (MUT-Mtmr10 and MUT-Msatl) (Fig. 2c). Interestingly, the average tumor volumes in the PBS and DC alone groups were 2133 ± 615 mm^3^ and 1983 ± 999 mm^3^, respectively. By contrast, the tumor volumes in the neoantigen-pulsed DC vaccine groups were as follows: 736 ± 243 mm^3^ in the MUT-Mtmr10 group, 791 ± 574 mm^3^ in the MUT-Elfn2 group, 559 ± 515 mm^3^ in the MUT-Kat8 group, 432 ± 422 mm^3^ in the MUT-Mastl group, 828 ± 837 mm^3^ in the MUT-Zscan21 group, and 1049 ± 1013 mm^3^ in the MUT-Mrpl1 group. The results indicate that all six neoantigen-pulsed DC vaccines significantly inhibited the tumor growth, compared to that of the PBS and non-DC-loaded groups. Of note, there was even no tumor growth in some mice treated with the neoantigen-pulsed DC vaccines (Fig. 2d, e). Moreover, treatments with 5/6 of the neoantigen-pulsed DC vaccines significantly increased the CD8^+^IFN-γ^+^ T-cell infiltration (Fig. 2f). Among the neoantigen-pulsed DC vaccines, the MUT-Mtmr10, MUT-Kat8, and MUT-Msatl vaccines exhibited the most evident inhibitory effects on tumor growth. These results demonstrated that 5/6 of the neoantigen-pulsed DC vaccines were effective, while only 2/6 of the neoantigen-adjuvant vaccines were effective in inhibiting LL2 xenograft growth, indicating that the antigen-pulsed DC vaccine had superior anti-tumor effects compared to neoantigen-adjuvant vaccines.

### Evaluation of additional immune responses between the neoantigen-pulsed DC vaccines and the neoantigen-adjuvant vaccines

**Fig. 3:**
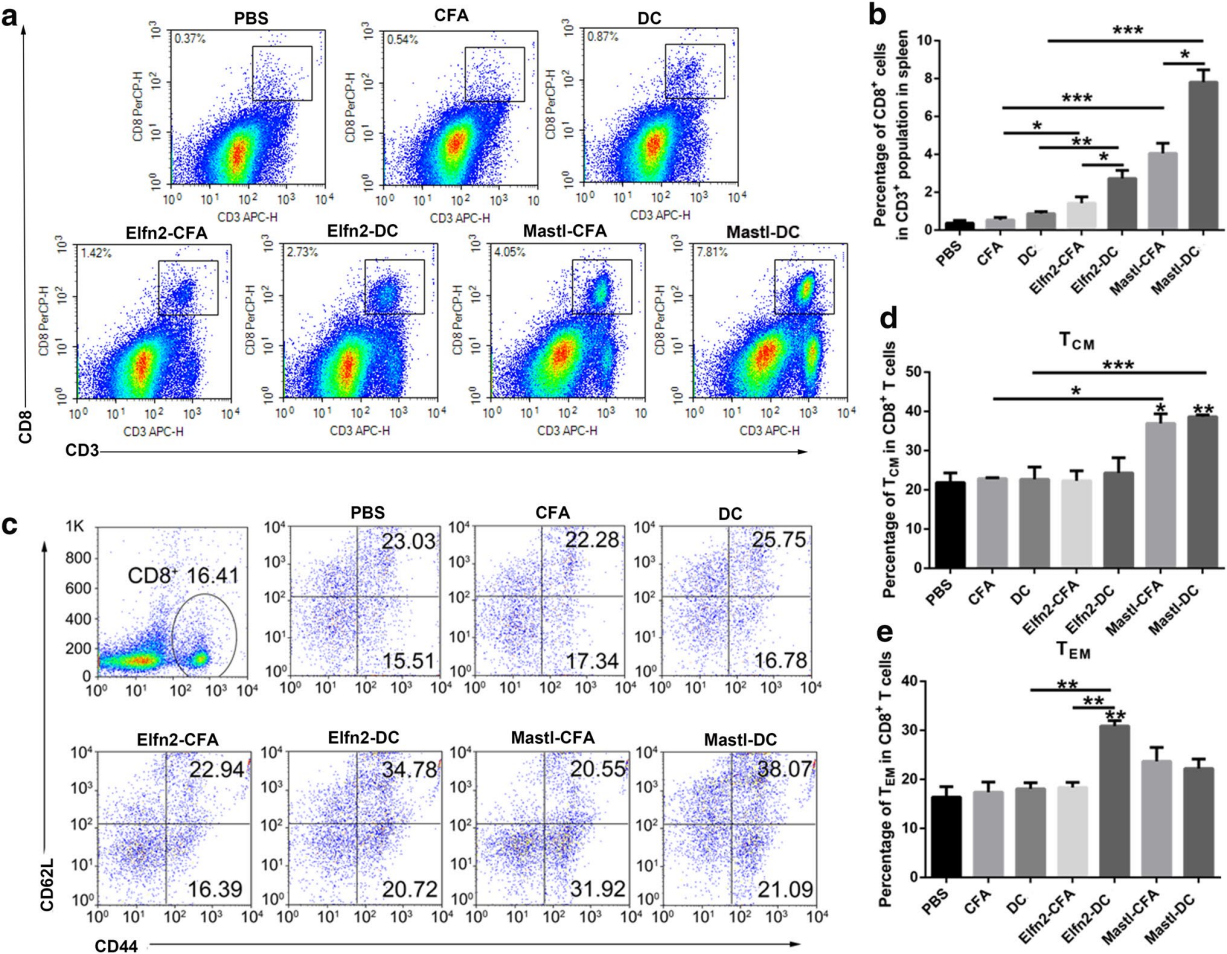
Effects of the neoantigen-adjuvant vaccines and the neoantigen-pulsed DC vaccines on memory T cells in the spleen. Mouse spleen lymphocyte cells were stained with anti-mouse-CD3, anti-mouse-CD8, anti-mouse-CD44, and anti-mouse-CD62L fluorescent antibodies, and then analyzed by flow cytometry. **a** Proportion of CD8^+^ T cells in the spleen. **b** Histogram of the percentage of CD8^+^ T cells in the spleen. **c** Proportions of CD44^+^ CD62L^+^ and CD44^+^ CD62L^−^ cells in the CD8^+^ T-cell population. **d**, **e** Statistical charts of the results of the CD8^+^ CD44^+^ CD62L^+^ T_CM_ (central memory T cells) population. **f** Statistical charts of the results of the CD8^+^ CD44^+^ CD62L^−^ T_EM_ (effector memory T cells) population. (*n* = 3). *P < 0.05, **P < 0.01 and ***P < 0.001

The goal of tumor immunization is to induce tumor-specific effector T-cell responses, thereby clearing the existing tumors and inducing immune memory responses to prevent tumor recurrence [24]. To gain more information about the differences in the immune responses between the neoantigen-pulsed DC vaccines and the neoantigen-adjuvant vaccines, we further evaluated the proportions of effector CD8^+^ T cells and memory CD8^+^ T cells in the spleen of mice treated with the two forms of vaccines. The results showed that the neoantigen-pulsed DC vaccine (MUT-Elfn2) significantly increased the proportion of CD8^+^ T cells in the spleen compared to that in the spleen of the mice treated with neoantigen-adjuvant vaccines (Fig. 3a, b). In addition, compared to the neoantigen-adjuvant vaccine, the neoantigen-pulsed DC vaccine also increased the proportion of CD8^+^CD44^+^CD62L^−^ cells (effector memory T cells, T_EM_) in the spleen (*P *< 0.01) (Fig. 3c–e). In addition, both the neoantigen-adjuvant vaccine and the neoantigen-pulsed DC vaccine with the MUT-Mastl neoantigen were able to significantly increased the proportion of CD8^+^CD44^+^CD62L^+^ cells (central memory T cells, T_CM_) (*P *< 0.05) (Fig. 3c–e). These results suggest that the neoantigen-pulsed DC vaccines activate stronger T-cell immune responses than the neoantigen-adjuvant vaccines.

**Fig. 4:**
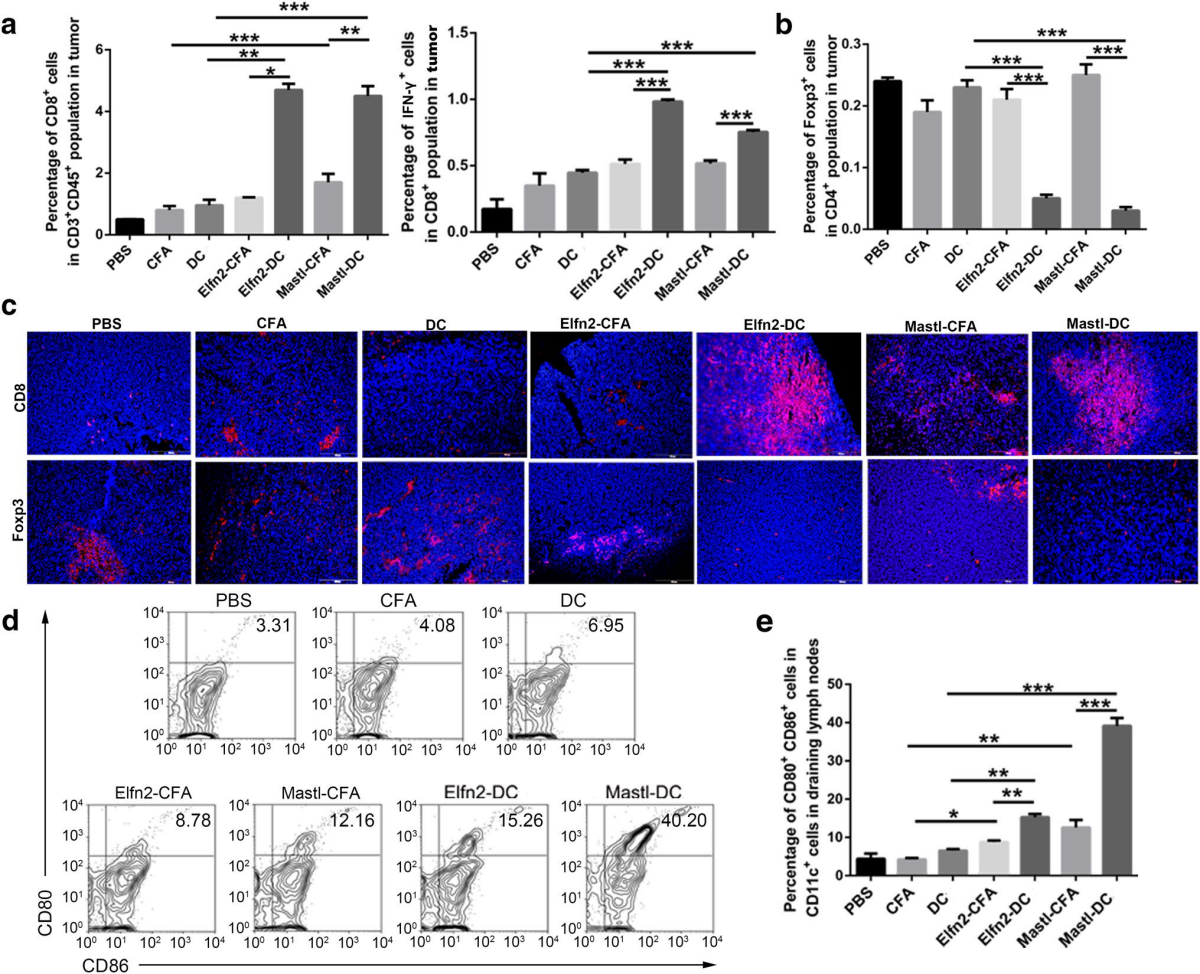
Effects of the neoantigen-adjuvant vaccines and the neoantigen-pulsed DC vaccines on T cells in tumor tissues and DCs in the tumor-draining lymph nodes. **a** Proportion of CD8^+^ and CD8^+^IFN-γ^+^ T cells in the CD45^+^ CD3^+^T-cell population in tumor tissues. **b** Proportion of Foxp3^+^ T cells in the CD4^+^ T-cell population in tumor tissues. **c** Expression levels of CD8 and Foxp3 in tumor tissues (× 200). **d** Proportion of CD80^+^ CD86^+^ cells in CD11c^+^ cells in the lymph nodes. **e** Histogram of the proportion of CD11c^+^ CD80^+^ CD86^+^ cells in the lymph nodes. (*n* = 3). *P < 0.05, **P < 0.01, and ***P < 0.001

The ratio of activated T cells to inhibitory T cells in the tumor microenvironment is an important parameter for evaluating the effect of cancer vaccines. Next, flow cytometry was used to determine the ratio of CD8^+^ and CD8^+^IFN-γ^+^ T cells in tumors from the mice immunized with the neoantigen-pulsed DC vaccines and the neoantigen-adjuvant vaccines. The results showed that compared to the neoantigen-adjuvant vaccines, the neoantigen-pulsed DC vaccines significantly increased the proportions of CD8^+^ and CD8^+^IFN-γ^+^ T cells in tumors (*P *< 0.05) (Fig. 4a). Flow cytometry also showed that the proportion of CD4^+^Foxp3^+^ cells was significantly decreased in the neoantigen-pulsed DC vaccine groups compared to the neoantigen-adjuvant vaccine groups (Fig. 4b). In addition, immunofluorescent staining showed that the vaccines facilitated CD8^+^ T-cell infiltration into the tumor tissue. Moreover, the neoantigen-pulsed DC vaccine groups had more CD8^+^ T cells and fewer Foxp3^+^ T cells infiltrating into the tumor microenvironment (Fig. 4c).

For the neoantigen-pulsed DC vaccines, the efficiency of DC migration to the lymph nodes is closely related to the effect of the vaccines. Neoantigen-loaded DCs migrate to lymph nodes and then present the antigens to T cells, thereby activating -ell responses [24]. For neoantigen-adjuvant vaccines, immature DCs in the lymph nodes need to be activated to develop into mature DCs, and then the mature DCs present their antigens to T cells, thus activating the T-cell response. In this study, MUT-Elfn2 and MUT-Mastl were used as two example peptides to study the effects of the neoantigen-pulsed DC vaccines and the neoantigen-adjuvant vaccines on the maturation of lymph-node DCs. The results showed that the expression levels of CD80 and CD86 on the surface of mature DCs were significantly higher in the neoantigen-pulsed DC vaccine groups and the neoantigen-adjuvant vaccine groups compared to the PBS, adjuvant and empty DC groups (Fig. 4d, e), suggesting that both the neoantigen-pulsed DC vaccines and the neoantigen-adjuvant vaccines could induce the transformation of DCs from immature to mature state. The results also revealed that the effect of the neoantigen-pulsed DC vaccines on lymph-node DC maturation was more efficient.

**Fig. 5:**
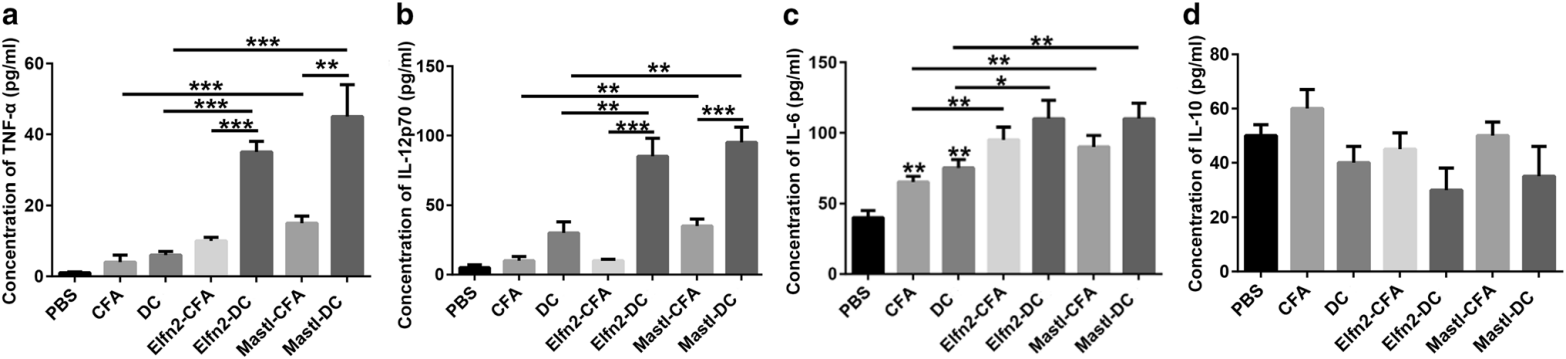
Effects of the neoantigen-adjuvant vaccines and the neoantigen-pulsed DC vaccines on the serum levels of cytokines. **a** Expression of TNF-α in the serum of mice immunized with the indicated antigen vaccines; **b** expression of IL-12p70 in the serum of mice immunized with the indicated antigen vaccines; **c** expression of IL-6 in the serum of mice immunized with the indicated antigen vaccines; **d** expression of IL-10 in the serum of mice immunized with the indicated antigen vaccines. *P < 0.05, **P < 0.01, and ***P < 0.001

IL-12p70 contributes to Th1 polarization of T cells [25]. IL-12p70 also promotes the secretion of TNF-α and IFN-γ, and participates in cytotoxic T-cell-mediated cellular immunity [25]. IL-6 can inhibit regulatory T cells, while IL-10 can inhibit cytotoxic immune cells [26, 27]. To understand why the neoantigen-pulsed DC vaccines display more potent immune responses than the neoantigen-adjuvant vaccines, we further examined the levels of the serum cytokines IL12p70, TNF-α, IL-6, and IL-10 in mice immunized with the two forms of vaccines. The ELISA results showed that the serum levels of TNF-α and IL-12p70 in the neoantigen-pulsed DC vaccine groups were significantly higher than those in the neoantigen-adjuvant vaccine groups (Fig. 5a, b). Also, the serum levels of IL-6 in the neoantigen-pulsed DC vaccine groups and the neoantigen-adjuvant vaccine groups were significantly higher than those in the control (PBS, adjuvant-alone, and empty DC) groups, despite no significant difference between the neoantigen-pulsed DC vaccine groups and the neoantigen-adjuvant vaccine groups (Fig. 5c). However, neither the neoantigen-pulsed DC vaccines nor the neoantigen-adjuvant vaccines significantly altered the serum levels of IL-10, compared to the controls (Fig. 5d). These data suggest that the neoantigen-pulsed DC vaccines induce stronger immune responses than the neoantigen-adjuvant vaccines possibly by upregulating the expression of TNF-α and IL-12p70 in the serum.

## Discussion

In this study, six MHC class I candidate neoantigens were screened in mouse LL2 lung cancer cell line. The neoantigen-adjuvant vaccines and the neoantigen-pulsed DC vaccines were prepared for individualized tumor treatments. On one hand, peptide vaccines have the advantages of simple synthesis, economical production, and clinical safety; on the other hand, peptide vaccines have two major shortcomings: low immunogenicity and MHC restriction [6]. Hence, currently, the most common solution is to add an immunological adjuvant, which is essential for inducing an effective immune response [28]. It is known that an adjuvant can (1) increase the biological half-life of the vaccines; (2) increase the antigen uptake by APCs; (3) promote the activation/maturation of APCs (i.e., DCs), inducing the production of immuno-regulatory cytokines; (4) activate inflammatory cells; and (5) induce local inflammation and cellular recruitment [29, 30]. In particular, Freund’s adjuvant is an oil-in-water emulsion that promotes the long-term retention and slow release of emulsified antigens at inoculation sites and can simultaneously produce Th1- and Th2-type immune responses. However, when Freund’s adjuvant is used in animals, severe ulceration occurs at the injection site. Due to the toxic side effects of many adjuvants, most of them cannot be used in human vaccine studies [31–33]. So far, aluminium adjuvants, such as Al(OH)_3_ and AlPO_4_, which primarily induce Th2 responses in therapeutic vaccines, are approved by the US-FDA for use in human vaccines [34]. Aluminium hydroxide needs to be combined with other adjuvants to induce CTL responses [35]. DCs are the most important and powerful APCs in vivo, for activating naïve T cells to exert specific immune responses. They take up, process, and present antigens and initiate and regulate immune responses [15]. DCs are a natural adjuvant and DC-based tumor vaccines are considered to be a promising anticancer agent [36]. Because of their safety and minimal side effects, DC-based tumor vaccines have been used in clinical trials for treatment of various tumors [37, 38].

There are significant differences in antigen presentation between antigen-adjuvant vaccines and antigen-pulsed DC vaccines. Adjuvants are non-specific immunopotentiators. When the antigen is injected or pre-injected into the body, the adjuvant can store the antigen, enhance the surface area of the antigen, prolong the retention time of the antigen in the body, and place the antigen in full contact with lymphocytes, thereby improving the efficiency of antigen presentation [7]. In addition, adjuvant vaccines can induce an inflammatory reaction at the injection site, allowing the immune cells to re-enter the injection site, thus increasing the efficiency of antigen uptake and presentation [39]. The adjuvant can also change the physical properties of the antigen by converting the soluble antigen into a solid state, facilitating phagocytosis of the antigen by APCs, hence improving the efficiency of ingestion and presentation [40]. Antigen-adjuvant vaccines activate CTL responses mainly by activating local APCs. When antigen-pulsed DC vaccines are prepared in vitro, the mature DCs loaded with antigens are returned to the patient from whom they were harvested. These cells migrate through the bloodstream to the secondary lymphoid tissues and directly present the antigen to the lymphocytes, activating the CTL response. Therefore, it is expected that antigen-pulsed DC vaccines should be more efficient than antigen-adjuvant vaccines with reference to activating immune responses and anti-tumor effects.

In this study, 4/6 of neoantigen-adjuvant vaccines, which contained MUT-Mtmr10, MUT-Elfn2, MUT-Msatl, and MUT-Zscan21 epitopes, induced significant neoantigen-specific splenic CD8^+^ T-cell responses. However, only 2/6 of the neoantigen-adjuvant vaccines (MUT-Mtmr10 and MUT-Msatl) were able to significantly inhibit tumor growth. Hailemichael and colleagues found that IFA-based peptide vaccines could induce potent CTL responses, but T-cell retention was negatively impacted, with T-cell depletion or loss occurring immediately after inoculation [41]. This is due to the long-term slow release of the emulsified antigen at the inoculation site, resulting in long-term antigen presentation, T-cell recognition, and cytokine release. Consequently, chronic inflammation and increased production of chemokines ensue, thus attracting and retaining effector T cells, which prevent them from reaching the tumor site. Based on these data, we further implemented a DC-based tumor immunotherapy strategy. There are fewer autologous DCs in tumor patients, because immune cells are usually immuno-suppressed or inactivated. Therefore, isolating and expanding enough DCs in vitro, then preparing DC-based vaccines loaded with neoantigens and administering them to patients for active immunotherapy have become a hot research direction in the field of cancer biotherapy. In this study, neoantigens were cultured with DCs, which were then injected intravenously into mice. In the LL2 tumor models, 6/6 of the neoantigen-pulsed DC vaccines induced a strong T-cell-specific response in spleen, and all neoantigen epitopes significantly elicited responses and inhibited tumor growth. Taken together, these results indicate that the neoantigen-pulsed DC vaccines can activate a stronger T-cell immune response and have a superior therapeutic effect to neoantigen-adjuvant vaccines.

The ultimate goal of a tumor vaccine is to either activate naive T cells, increase effector T cells, or induce memory T cells to achieve tumor therapies [24]. In this study, we found that compared to the neoantigen-adjuvant vaccines, the neoantigen-pulsed DC vaccines not only increased the number of central memory T cells and effector memory T cells, but also induced effector CD8^+^ T cells to secret IFN-γ. Also, there were significantly more activated T cells and significantly fewer immunosuppressive regulatory T cells in the tumor tissues of the antigen-pulsed DC vaccine groups. Therefore, our findings support that the neoantigen-pulsed DC vaccine is a promising approach for cancer immunotherapy.

## Abbreviations

CFA: Complete Freund’s adjuvant
IFA: Incomplete Freund’s adjuvant
IFN-γ: Interferon gamma
IL-6: Interleukin-6
IL-10: Interleukin-10
IL-12: Interleukin-12
MAGE: Melanoma-associated antigen
MUT: Mutant
NGS: Next-generation sequencing
PS: Penicillin/streptomycin
T_CM_: Central memory T cells
T_EM_: Effector memory T cells
TNF-α: Tumor necrosis factor-α
WT: Wild type

## References

[1] Castle J, Kreiter S, Diekmann J (2012). Exploiting the mutanome for tumor vaccination. Ann Oncol.

[2] Yadav M, Jhunjhunwala S, Phung QT (2014). Predicting immunogenic tumour mutations by combining mass spectrometry and exome sequencing. Nature.

[3] Simon R, Roychowdhury S (2013). Implementing personalized cancer genomics in clinical trials. Nat Rev Drug Discov.

[4] Ogi C, Aruga A (2015). Approaches to improve development methods for therapeutic cancer vaccines. Immunol Lett.

[5] Vergati M, Intrivici C, Huen NY, Schlom J, Tsang KY (2010). Strategies for cancer vaccine development. J Biomed Biotechnol.

[6] Wang X, Li X, Yoshiyuki K, Watanabe Y, Sogo Y, Ohno T, Tsuji NM, Ito A (2016). Cancer immunotherapy: comprehensive mechanism analysis of mesoporous-silica-nanoparticle-induced cancer immunotherapy. Adv Healthc Mater.

[7] Awate S, Babiuk LA, Mutwiri G (2013). Mechanisms of action of adjuvants. Front Immunol.

[8] Rooney MS, Shukla SA, Wu CJ, Getz G, Hacohen N (2015). Molecular and genetic properties of tumors associated with local immune cytolytic activity. Cell.

[9] Matsushita H, Vesely MD, Koboldt DC (2012). Cancer exome analysis reveals a T-cell-dependent mechanism of cancer immunoediting. Nature.

[10] Li AW, Sobral MC, Badrinath S (2018). A facile approach to enhance antigen response for personalized cancer vaccination. Nat Mater.

[11] Kuai R, Ochyl LJ, Bahjat KS, Schwendeman A, Moon JJ (2017). Designer vaccine nanodiscs for personalized cancer immunotherapy. Nat Mater.

[12] Xia Y, Wu J, Wei W (2018). Exploiting the pliability and lateral mobility of Pickering emulsion for enhanced vaccination. Nat Mater.

[13] Xiang J, Xu L, Gong H (2015). Antigen-loaded upconversion nanoparticles for dendritic cell stimulation, tracking, and vaccination in dendritic cell-based immunotherapy. ACS Nano.

[14] Brossart P, Wirths S, Stuhler G, Reichardt VL, Kanz L, Brugger W (2000). Induction of cytotoxic T-lymphocyte responses in vivo after vaccinations with peptide-pulsed dendritic cells. Blood.

[15] Constantino J, Gomes C, Falcao A, Cruz MT, Neves BM (2016). Antitumor dendritic cell-based vaccines: lessons from 20 years of clinical trials and future perspectives. Transl Res.

[16] Madan RA, Gulley JL, Fojo T, Dahut WL (2010). Therapeutic cancer vaccines in prostate cancer: the paradox of improved survival without changes in time to progression. Oncologist.

[17] Phuphanich S, Wheeler CJ, Rudnick JD (2013). Phase I trial of a multi-epitope-pulsed dendritic cell vaccine for patients with newly diagnosed glioblastoma. Cancer Immunol Immunother.

[18] Steven A, Rosenberg JCY, Restifo NP (2004). Cancer immunotherapy: moving beyond current vaccines. Nat Med.

[19] Li H, Durbin R (2009). Fast and accurate short read alignment with Burrows-Wheeler transform. Bioinformatics.

[20] Cibulskis K, Lawrence MS, Carter SL (2013). Sensitive detection of somatic point mutations in impure and heterogeneous cancer samples. Nat Biotechnol.

[21] Koboldt DC, Chen K, Wylie T, Larson DE, McLellan MD, Mardis ER, Weinstock GM, Wilson RK, Ding L (2009). VarScan: variant detection in massively parallel sequencing of individual and pooled samples. Bioinformatics.

[22] Wang K, Li M, Hakonarson H (2010). ANNOVAR: functional annotation of genetic variants from high-throughput sequencing data. Nucleic Acids Res.

[23] Liu G, Li D, Li Z (2017). PSSMHCpan: a novel PSSM-based software for predicting class I peptide-HLA binding affinity. GigaScience..

[24] Palucka K, Banchereau J (2012). Cancer immunotherapy via dendritic cells. Nat Rev Cancer.

[25] Trinchieri G (2003). Interleukin-12 and the regulation of innate resistance and adaptive immunity. Nat Rev Immunol.

[26] Fujimoto M, Nakano M, Terabe F (2011). The influence of excessive IL-6 production in vivo on the development and function of Foxp3(+) regulatory T cells. J Immunol.

[27] Skalova K, Mollova K, Michalek J (2010). Human myeloid dendritic cells for cancer therapy: does maturation matter?. Vaccine..

[28] Apostolico Jde S, Lunardelli VA, Coirada FC, Boscardin SB, Rosa DS (2016). Adjuvants: classification, modus operandi, and licensing. J Immunol Res.

[29] Di Pasquale A, Preiss S, Tavares Da Silva F, Garcon N (2015). Vaccine adjuvants: from 1920 to 2015 and beyond. Vaccines..

[30] Coffman RL, Sher A, Seder RA (2010). Vaccine adjuvants: putting innate immunity to work. Immunity.

[31] Reinhardt RL, Bullard DC, Weaver CT, Jenkins MK (2003). Preferential accumulation of antigen-specific effector CD4 T cells at an antigen injection site involves CD62E-dependent migration but not local proliferation. J Exp Med.

[32] Redmond WL, Sherman LA (2005). Peripheral tolerance of CD8 T lymphocytes. Immunity.

[33] Alving CR, Matyas GR, Torres O, Jalah R, Beck Z (2014). Adjuvants for vaccines to drugs of abuse and addiction. Vaccine..

[34] Baylor NW, Egan W, Richman P (2002). Aluminum salts in vaccines–US perspective. Vaccine..

[35] Khong H, Overwijk WW (2016). Adjuvants for peptide-based cancer vaccines. J Immunother Cancer..

[36] Sabado RL, Bhardwaj N (2015). Cancer immunotherapy: dendritic-cell vaccines on the move. Nature.

[37] Bloy N, Pol J, Aranda F (2014). Trial watch: dendritic cell-based anticancer therapy. Oncoimmunology..

[38] Makkouk A, Weiner GJ (2015). Cancer immunotherapy and breaking immune tolerance: new approaches to an old challenge. Cancer Res.

[39] Mutoloki S, Alexandersen S, Gravningen K, Evensen O (2008). Time-course study of injection site inflammatory reactions following intraperitoneal injection of Atlantic cod (*Gadus morhua* L.) with oil-adjuvanted vaccines. Fish Shellfish Immun..

[40] Lambrecht BN, Kool M, Willart MAM, Hammad H (2009). Mechanism of action of clinically approved adjuvants. Curr Opin Immunol.

[41] Hailemichael Y, Dai ZM, Jaffarzad N (2013). Persistent antigen at vaccination sites induces tumor-specific CD8(+) T cell sequestration, dysfunction and deletion. Nat Med.

